# New Organophilic Montmorillonites with Lactic Acid Oligomers and Other Environmentally Friendly Compounds and Their Effect on Mechanical Properties of Polylactide (PLA)

**DOI:** 10.3390/ma14216286

**Published:** 2021-10-21

**Authors:** Katarzyna Rucińska, Zbigniew Florjańczyk, Maciej Dębowski, Tomasz Gołofit, Rafał Malinowski

**Affiliations:** 1 Łukasiewicz Research Network—Institute for Engineering of Polymer Materials and Dyes, M. Skłodowska-Curie 55, 87-100 Toruń, Poland; 2 Faculty of Chemistry, Warsaw University of Technology, Noakowskiego 3, 00-664 Warsaw, Poland; evala@ch.pw.edu.pl (Z.F.); maciej.debowski@pw.edu.pl (M.D.); tomgol@ch.pw.edu.pl (T.G.)

**Keywords:** montmorillonite, organophilization, lactic acid oligomers, intercalation, X-ray diffraction, PLA

## Abstract

New organophilic montmorillonites with oligomers of lactic acid and other compounds such as citric acid, stearic acid, maleic anhydride, pentaerythritol and ε-caprolactone were synthesized. They were characterized by Fourier Transform Infrared Spectroscopy (FTIR), X-ray Diffraction (XRD), Thermogravimetric Analysis (TGA), Scanning Electron Microscopy (SEM), elemental analysis and swelling capacity in water. In all tested composites, an increase in the montmorillonite interlayer distance resulting from intercalation of the modifying substance in the montmorillonite was found by means of XRD. Elemental analysis and FTIR showed that all of the tested samples contained an organic segment in the montmorillonite structure. TGA studies revealed that composites modified with lactic acid oligomers, stearic acid or ε-caprolactone had the highest thermal stability. They also exhibited the lowest swelling capacity which was 2–3 times lower than that for unmodified sodium montmorillonite. Some preliminary studies on the mechanical properties of PLA/modified montmorillonite are also presented and discussed.

## 1. Introduction

Sodium montmorillonite (Mt) belongs to a group of natural aluminosilicates with a 2:1 layered structure [1]. It is the main component of bentonite, a clay mineral derived from the transformation of volcanic ash. The crystal structure of Mt consists of three layers connected by a common oxygen atom. Two outer layers with a tetrahedral silicon dioxide crystal structure and one internal layer consisting of octahedral crystals of magnesium oxide or aluminum oxide form a package with gap between them called the interlayer spacing or gallery [2]. Isomorphic substitution within the layer (for example Al^3+^ replaced by Fe^2+^) generates negative charges that are counterbalanced by sodium cations located in the gallery. The existence of interlayer cations gives them the ability to absorb water, as a way to increase the distance between the Mt layers. Montmorillonite is characterized by high water absorption, it can absorb five times more water than it weighs, simultaneously increasing its volume up to fifteen times [3,4]. The interlayer spacing can be modified by introducing different cations or molecules into their structure. These capabilities are utilized by introducing various inorganic cations between the Mt layers, which are used as catalytic layer precursors [5] or organic cations, most often derived from quaternary ammonium salts [6].

The greatest practical application of Mt modifiers is that they have cationic compounds containing at least one long alkyl chain in their molecules. Some good example of them include derivatives of octadecylamine, as well as salts obtained from a mixture of amines collected from the chemical processing of fatty acids [7,8,9,10]. The presence of long alkyl chains in the interlayer space repeals water from it and increases the interlayer distance. Such modification, known as organophilization, favors the creation of intercalated or exfoliated composites. The susceptibility of ammonium salts containing alkyl chains to thermal decomposition leading to the elimination of olefins is considered to be the main disadvantage of their use as Mt modifiers [11,12]. To improve the stability of organophilic montmorillonites, other types of organic cations have been used, e.g., phosphonium, imidazolium, or aryl ammonium [13,14,15]. However, this leads to a significant increase in the cost of modified Mt. Organically modified aluminosilicates have been extensively tested and are used as components of composites [16,17,18,19], as organic molecules adsorbents in several water-soil or air treatment technologies, as well, as rheology modifiers in paints, cosmetics, lubricants, and oil-based drilling fluids [20,21,22,23,24].

Organophilized montmorillonite (OMt) has also been applied in the modification of the properties of polylactide (PLA). De Tarso Vieira Erosa et al. [25] carried out a study on the thermal stability and fire properties of PLA nanocomposites with 2, 6, and 8 wt.%, single and hybrid Brazilian OMt containing ammonium- and phosphonium- based surfactants such as dimethyl di-(hydrogenated tallow) ammonium chloride (HTA), trihexyl tetradecyl phosphoniu chloride (TDP), di(alkyl ester) dimethyl ammonium chloride (EA) and ethoxylated tallow amine (ETA). The addition of OMt and extrusion resulted in the reduction of the initial decomposition temperature and average molecular weight of PLA, as assessed by thermogravimetric analysis (TGA) and gel permeation chromatography (GPC), respectively. In addition, PLA nanocomposites with single OMt (TDP and HTA) were less thermally stable than nanocomposites with hybrid OMt.

In a study by Bezerra Lima et.al. [26], the effects of concentration and surface modification of two Brazilian bentonite clays on the properties of PLA based nanocomposites were investigated. Samples of biodegradable packing plastics were prepared by extrusion/injection molding and characterized by different techniques. The natural clay particles were approximately 2μm in size, while those present in the modified clay samples were larger (5–6 μm in size), probably due to the presence of cetyltrimethylammonium bromide in the interlayer space. The addition of both organic clays to PLA decreased its elongation at break and tensile strength of the sample, while increasing the Young’s modulus. In a study by [27], PLA nanocomposites based on single and hybrid OMt were investigated for morphological, thermal, and fire performance. Flexibility and impact strength studies showed that hybrid OMt containing ammonium ester and ethoxylated amine improved the plasticity, ductility, and impact strength of PLA. This behavior was explained by the high level of compatibility and interaction between surfactants and PLA chains due to the presence of polar groups in their structure.

Bijarimi et al. [28] described the preparation and characterization of polylactic acid/acrylonitrile-butadiene-styrene/graphene and HTA-functionalized clay (C20A montmorillonite) in melt. The clay was combined with graphene to improve its dispersion in the polymer matrix. The properties of the PLA/ABS/GnP/C20A nanocomposites depended significantly on the processing temperature, which dictated the degree of thermal stability and filler dispersion. The hybrid nanocomposites were characterized in terms of their stress-strain, thermal, chemical, and morphological properties. An increase in tensile strength and Young’s modulus was found for PLA/ABS/GnP/C20A in the high temperature profile.

The aim of this study was to find new greener ways to modify Mt. We have attempted to replace thermally unstable and toxic compounds currently used to make Mt more organophilic with lactic acid oligomers and their derivatives with other, new organic compounds that are environmentally friendly. The results of some preliminary studies on the mechanical properties of PLA containing these new compounds will be also discussed.

## 2. Materials and Methods

### 2.1. Materials

The study used unmodified sodium montmorillonite, Dellite HPS, by LaviosaChimicaMineraria Spa, (Mt), used without prior purification, characterized as high CEC, L −(+)− lactic acid 85% water solution, (LAc), stearic acid (95%), (SAc), citric acid (99%), (CAc), pentaerythritol (99%), (PT), ε-caprolactone (97%), (CAP) from Sigma Aldrich, and maleic anhydride (99.8%), (MA) from POCH. All reagents were used without purification. Polylactide, Ingeo2003D from NatureWorks LLC was used as the polymer matrix.

### 2.2. Modification of Sodium Montmorillonite

The modification of montmorillonite was carried out in a two-necked round-bottomed flask (100 cm^3^) (LABIT, Warsaw, Poland), equipped with a magnetic stirring element. A total of 44.32 g of −(+)− lactic acid (LAc) was weighed and 4.32 g of montmorillonite was introduced, then the flask was heated in an oil bath. After placing the reactants in the reactor, the reaction mixture was gradually heated to 170 °C within 1 h and left at this temperature for another 4 h, while being vigorously stirred. In the next step, the contents of the flask were cooled to 100 °C, and then the pressure was reduced to 600 mbar. The reaction was carried out in this way for another 4 h, once more gradually increasing the temperature to 170 °C. After completing the reaction and cooling the system to room temperature, the crude product was suspended in chloroform and the resulting suspension was filtered on a Buchner funnel (LABIT, Warsaw, Poland). The precipitate was washed with the help of centrifugation and filtered several times with fresh solvent and dried under atmospheric pressure. 4.20 g of a gray solid was obtained, which was a montmorillonite composite with an L-lactic acid oligomer (product abbreviated Mt-LAc). The same procedure, excluding the addition of Mt, was used to obtain a pure LAc oligomer that was subsequently applied in the reactions with SAc and CAP. 

In some experiments a third reactant containing reactive hydroxyl or carboxyl groups was added to the mixture of LAc and Mt. The molar ratio of this additional substrate to LAc was as follows: 1:15 for citric acid (product abbreviated as Mt-LAc-CAc), 1:20 for pentaerythritol (product abbreviated as Mt-LAc-PT), or 1:2 for maleic anhydride (product abbreviated as Mt-LAc-MA).

In order to obtain OMt containing highly hydrophobic stearyl groups (product abbreviated as Mt-LAc-SAc) a separately synthesized LAc oligomer was reacted with stearic acid in the presence of Mt. The weight ratio of the LAc oligomer to SAc: and Mt was 10:5:1, respectively. The process was carried out at 170 °C for 8 h. Separation and purification of the product were carried out under the same conditions as during Mt-LAc synthesis.

OMt containing fragments of CAP was synthesized from ε-caprolactone, 29.0 g, and unpurified Mt with lactic acid oligomer, 18.5 g, (product abbreviated as Mt-LAc-CAP). The raw materials were mixed for about 10 h at 170 °C. The obtained modified Mt precipitate was purified in the same way as the primary version of modification with a lactic acid oligomer.

For clarity of presentation, the applied synthetic procedures are also expressed in the form of process flow charts depicted in Figures S1–S5 in the Electronic Supporting Information.

### 2.3. Obtaining a PLA Composite with Selected Modified Montmorillonites

Preliminary investigations were carried out for lines samples extruded in a Thermo HaakeMiniLab II twin-screw laboratory extruder equipped with a return channel and operating in a counter-rotating system. PLA without Mt, PLA with unmodified Mt sodium, and PLA with Mt modified with lactic acid oligomers and poly(ε-caprolactone) were obtained. A 5 wt. % filler was used in relation to PLA. Composites components weighed on a balance (7g weight) (RADWAG, Radom, Poland) were introduced into the device in 3 portions. Dosing of the components of the composites and their homogenization were carried out at 180 °C for 10 min at a screw speed of 50 r/min. After this time, the screw speed was reduced to 20 r/min and the composite was extruded in the form of a cord using a mouthpiece with an opening diameter of 0.5 mm. Samples of 8 cm length were obtained from the resulting strands and 10 samples of each type of similar weight were selected.

### 2.4. Test Methods and Apparatus

- The carbon and hydrogen content of the samples was determined using a Perkin Elmer CHNS/O II 2400 instrument (Perkin Elmer, Waltham, MA, USA).
- Fourier transform infrared spectra were recorded on a Perkin-Elmer Paragon 1000 spectrometer (Perkin Elmer, Waltham, MA, USA) equipped with a PIKE MIRacle ATR attachment with an AMTIR crystal from Pike Technologies. The wave number range from 500 to 4000 cm^−1^ was used with automatic background correction. Measurement control and data acquisition were performed using Perkin-Elmer’s Spectrum^®^ 5.3 software.
- The XRD measurements of the powder samples were carried out on a D8 Discover diffractometer (Bruker, Billerica, MA, USA) equipped with a ceramic X-ray tube with a copper anode (radiation wavelength 1.54 Å). The diffractometer operates with a parallel beam system, a monochromator—a Goebel mirror (parabolic), and a position sensitive Vantec 1 detector. The tests were performed at room temperature.
- Thermogravimetric analysis was performed using the SDT Q600 V20.9 Build 23 instrument (TA Instruments, New Castle, DE, USA) from TA Instruments. Measurements were made in air or nitrogen atmospheres in the temperature range of 25–800 °C, the heating rate was 20 °C/min.
- The morphology of the samples was analyzed using an ULTRA Plus field emission scanning electron microscope (Zeiss, Jena, Germany) with a Zeiss GEMINI column (Zeiss, Jena, Germany), equipped with 2 secondary electron detectors: a standard one in the SE2 chamber and an in-column InLens. Prior to SEM measurement, the samples were vacuum sprayed with a conductive carbon layer.
- The swelling capacity was measured at room temperature by immersing a sample of dry powder weighing about 0.25 g in 100 cm^3^ of deionized water for 72 h. The samples were pre-dried at 110 °C for 3 h. After equilibrium was reached, excess water was removed from the sample by filtration and the material was dried to a constant weight. The swelling capacity (S_eq_) was determined gravimetrically using the following equation:
  (1) $$S_{\mathrm{eq}}=\frac{m_{\mathrm{wet}}-m_{\mathrm{dry}}}{m_{\mathrm{dry}}}=\frac{m_{\mathrm{water}}}{m_{\mathrm{dry}}}$$
  where: S_eq_—swelling capacity, m_wet_—mass of wet sample, m_dry_—mass of dried sample, m_water_—mass of absorbed water.
- Apparatus for strength measurements—Instron 5566 (Instron, Norwood, MA, United States). Measurements of mechanical parameters of rigid or flexible materials in the tensile or bending mode; the possibility of measuring samples of various shapes (foil, line/cord, bar).

## 3. Results and Discussion

In order to obtain organophilic montmorillonites with lactic acid oligomers and other compounds, condensation reactions of lactic acid in the presence of sodium montmorillonite were performed, as well as reactions of lactic acid with other compounds of hydrophilic or hydrophobic nature. Because one could expect that larger LAc oligomers should encounter difficulties integrating into the structure of Mt, we selected such processing conditions (e.g., molar ratio of reagents, time and pressure on each stage of the process), as to promote the formation of small LAc oligomers having an average molecular mass less than 1000 g/mol. Our previous studies on the LAc copolymers [29,30,31,32] indicate that, under such processing conditions, condensation of LAc in the absence of Mt produces LAc oligomers with molar masses 500–1000 g/mol (reactions with CAc, PT, or without additional reagent), or 260 g/mol (condensation with MA).

### 3.1. Elemental Analyses

The results of the elemental analysis tests are presented in Table 1. They showed that all tested samples contained an organic segment in the Mt structure. The raw, sodium Mt contained 0.9% wt. carbon and 1.56% wt. of hydrogen, while the Mt modified with lactic acid oligomers (Mt-LAc) and other compounds contained from 12.79% to 21.43% wt. carbon and 2.05% to 3.37% wt. hydrogen. The composite, where the modifying substance was the reaction product of lactic and citric acid (Mt-LAc-CAc), contained 13.3% wt. carbon and it was about 3–4% less than in the case of modification with the lactic acid oligomer alone. The Mt-LAc-PT contained 14.8% wt. carbon and 2.25% wt. hydrogen. In the case of montmorillonite modified with the reaction product of lactic acid and maleic anhydride (Mt-LAc-MA), the carbon content in the isolated solid product was 21.4% by weight, which, assuming that the chains incorporated in the mineral have a composition similar to the expected one, allowed us to estimate that the organic part accounted for about 46% wt. of the composite. This was distinctly more than in the other previously described composites. The carbon content of the Mt-LAc-CAP was 19.99% wt. and likewise it was one of the highest values. The isolated composite from Mt-LAc-SAc contained on average 12.8% wt. carbon and it was the lowest value of all modified Mt. This could be due to the fact that stearic acid was the most hydrophobic among the modifying substances, and due to the reaction taking place in an aqueous environment, it had the lowest possibility of being incorporated into the Mt structures.

### 3.2. FTIR Spectroscopy of Sodium Mt and Organophilic Montmorillonites

**Figure 1:**
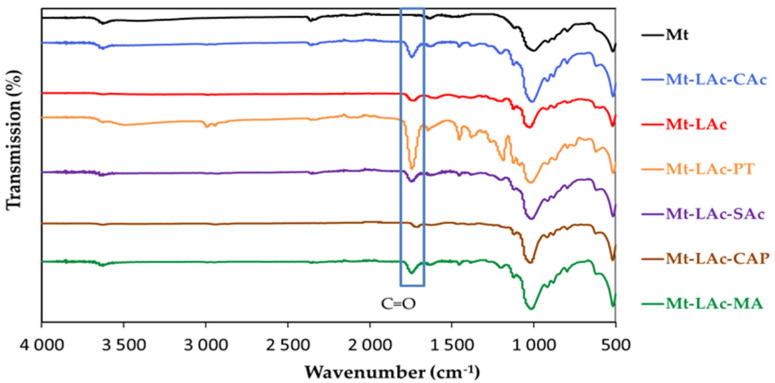
FTIR spectra for Mt and organophilic montmorillonites.

Infrared spectroscopy was used to analyze the structure of Mt and its modified versions. The spectrum of sodium montmorillonite (labelled as Mt) indicated the presence of water and contained bands characteristic of OH groups in aluminosilicate. In addition to signals from Mt, characteristic signals of the modifying substances also appeared. Figure 1 shows the Mt spectrum and the modified Mt spectra, where absorption bands characteristic of OH groups are observed (stretching vibrations at the wave number 3400–3700 cm^−1^ and bending vibrations at 1600–1700 cm^−1^). A band of bending vibrations for Mt characteristic of Al-OH groups at 912 cm^−1^ was also observed, as well as bands of stretching vibrations of Si-O bonds at 1123 cm^−1^ and 1002 cm^−1^ and for the same bond a band of bending vibrations at 529 cm^−1^. For all samples, in particular Mt-LAc-PT and Mt-LAc-SAc, there was the existence of a band in the range of 2850–3000 cm^−1^ that can be attributed to the stretching vibrations of CH bonds. Bands seen in the range of 1100–1300 cm^−1^ correlate to bending vibrations of CH. There are also visible characteristic bands for aliphatic esters: stretching vibrations of C = O and C-O bonds, at approx. 1745 and 1195 cm^−1^, respectively. For the Mt-LAc-PT sample, in particular, due to the structure of pentaerythritol, there were characteristic -OH bending vibration bands in the range of 1330–1420 cm^−1^ and 650–770 cm^−1^. For all tested modified Mt, a narrowing of the peak at 1000 cm^−1^ was observed. This may have indicated that the vibrations from Si-O had ceased to overlap, and thus resulted in a widening of the layers in Mt. This was confirmed by XRD analysis.

### 3.3. X-ray Diffraction of Modified Montmorillonites

**Figure 2:**
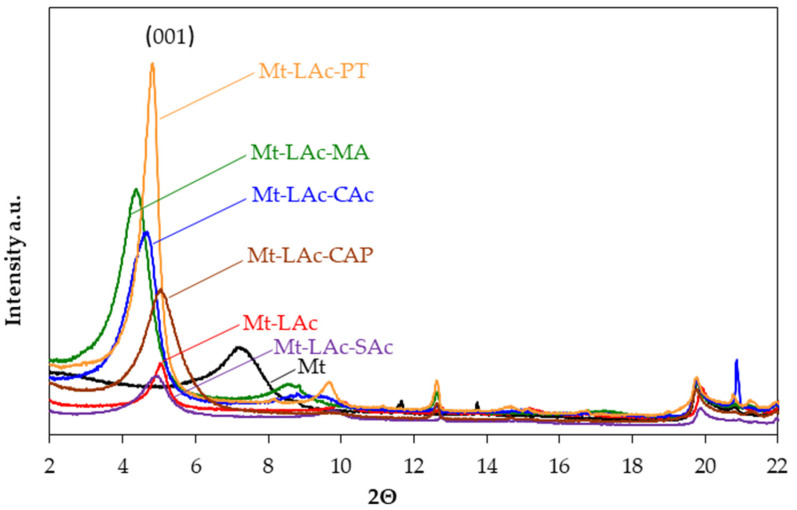
XRD diffractogram of sodium Mt and organophilic montmorillonites.

X-ray diffraction tests were carried out on the unmodified sodium Mt, (Figure 2, Table 1), the peak 001 was observed, which is related to the arrangement of the aluminosilicate layers occurring at the value of the angle 2Θ approx. 7.50°; this corresponded to the interlayer distance (d_001_) of 1.28 nm. For the modified organophilic montmorillonites, the values of the interlayer distances were in the range of 1.75 to 2.01 nm a sign of the separation of layers. It was observed that after modification, for all modified montmorillonites, the peak (*001*) shifted towards the lower value of the angle 2Θ, which meant that the distance between the layers was increased. For Mt-LAc, the d_001_ parameter values ranged from 1.74–1.77 nm, so they were about 0.5 nm bigger than in the starting sodium Mt material. It was found that for Mt-LAc-CAc, the organic material was also incorporated between the Mt layers, and the value of the d_001_ parameter (1.91 nm) was slightly bigger than in composites with unmodified composite. The position of the other peaks did not change significantly. Mt-LAc-PT diffraction tests showed that the parameter d_001_ for these products was 1.83 nm, so the distance between the layers was slightly larger than in composites with linear oligomers. It could also probably be related to the presence of more water in the space between the mineral layers. In addition, the was no significant change in the value of the parameter d_001_ (1.79 nm) for montmorillonites modified with the reaction products of lactic acid and stearic acid. However, additional sharp peaks did appear on the diffractogram, which could have come from the crystalline phase formed by free or incorporated stearic acid. The d_001_ value for Mt-LAc-CAP was 1.75 nm and the interlayer distances did not change significantly compared to Mt-LAc. On the contrary, the diffractogram analysis for Mt-LAc-MA showed that the value of the parameter d_001_ was 2.01 nm and it was the highest value among all tested composites. This was probably due to the high organic material content in the spaces between the layers and the high polarity of the short chains with terminal carboxyl groups, which promoted water absorption.

### 3.4. Thermal Stability of Modified Montmorillonites

**Figure 3:**
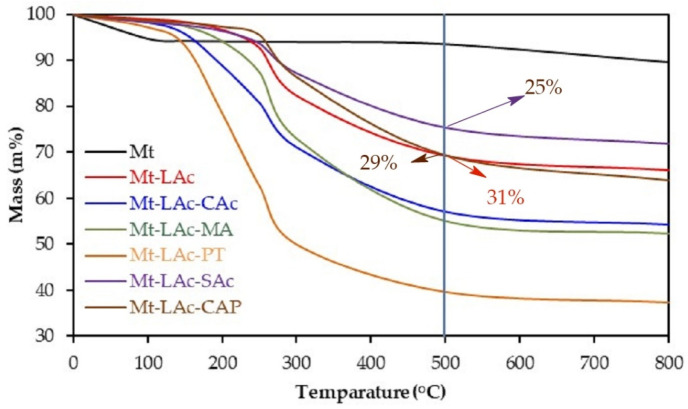
Mass loss as a function of temperature for modified Mt samples.

TGA analysis—Figure 3, showed that the Mt samples stored and annealed in air contained about 5.8% absorbed water. Heating to around 180 °C removed this water. The water could be bound to both the surface of the samples and in the spaces between the layers, its total content depended on the water vapor content in the air. In total, during annealing to 800 °C, the mass of the samples was reduced by 10–11% wt.

During annealing of the lactic acid oligomers at an elevated temperature, apart from condensation, depolymerization also occurred, this resulted in the elimination of the lactide. It was found that such a process also worked on the obtained composites. As a result of annealing for 10 h at 180 °C under atmospheric pressure, in samples containing about 34%wt. of organic material, the carbon content dropped from 17.5% to 12.1%wt. Thermogravimetric analyses of Mt-LAc showed that, during annealing up to 500 °C, they lost 31–38% of their mass—Figure 3. It could be assumed with high probability that in this temperature range all organic matter and water absorbed were removed from the composite as a result of progressive polycondensation. The amount of absorbed water, which was removed mainly during annealing up to 100 °C was 1–2% and was distinctly lower than in the solely Mt samples. This helped to confirm the conclusion drawn from the observation of FTIR spectra about the reduction of the hydrophilic properties of Mt after introducing lactic acid oligomers into its structure. It’s likely that a small amount of the absorbed water was also released at the higher temperatures. Although it can be assumed that in the range of 100–500 °C the main processes that took place were condensation with water separation, lactide elimination, and thermal decomposition of the remaining oligomers. The total weight loss in this range (30–36% wt.) was consistent with the expected organic material content determined on the basis of the carbon content. The weight loss temperature profiles for different samples with different organic material content were different, which may have been because some of the chains were absorbed outside of the Mt particles and degraded earlier than the chains between the mineral layers, which delayed the flow of heat.

As a result of heating to 800 °C, the Mt-LAc-SAc composite lost 28.2% of its weight, of which about 25% at this temperature range is typical for the decomposition of organic matter. In the range of 100 to 200 °C, the sample lost about 1.9% of its weight. It was one of the lower figures for this study. Thermogravimetric analysis of Mt-LAc-CAc showed that during annealing up to 800 °C, it lost nearly 46% of its weight, of which about 43% was in the temperature range of 100–500 °C. Thermogravimetric analyses for Mt-LAc-PT showed that in the range of room temperature to 800 °C, the samples of this composite lost about 63% of their weight, i.e., more than the samples modified with linear oligomers of lactic acid (~40%). This can only be explained by the greater tendency of composites containing pentaerythritol derivatives to absorb water. The analysis of weight loss as a function of temperature indicated that, under the measurement conditions, the ester bonds were probably hydrolyzed. This is because the weight loss due to water desorption at temperatures up to 150 °C was only slightly greater than in the samples of unmodified Mt. While the weight loss in the temperature range of 150–250 °C was definitely higher than in composites with linear oligomers (over 30%). It was found that lactic acid and pentaerythritol can be released in this temperature range. An additional reason for the large weight loss could be the faster secretion of lactide due to the increased concentration of the hydroxyl end groups that are active in backbiting processes. The total weight loss during annealing up to 800 °C for the Mt-LAc-MA sample was about 48%, of which about 2.5% was water. During standard annealing at 100 °C the sample lost about 6.5%. The water absorption capacity of this sample (~3.1 g/g) was slightly higher than those for composites with pentaerythritol, but still significantly lower than that for unmodified Mt. The thermogravimetric analysis of the Mt-LAc-CAP sample showed that the total weight loss during annealing up to 800 °C was about 36%, of which, in the range of 150–500 °C—typical for decomposition of the organic phase, the sample lost about 29% of its mass. Analysis on a sample of the starting composite showed a loss of about 34% wt. in this same range. This data provided some indication that both poly(lactic acid) and poly(ε-caprolactone) segments were present in the starting composite. It was also very interesting to compare the weight loss in the range from 150 to 250 °C, i.e., in the range typical for processing of thermoplastics. Samples containing only lactic acid oligomers lost 6–12% wt. in this range, whereas samples containing poly(ε-caprolactone) lost about 2.5%. This led to the conclusion that, in the latter samples, condensation and depolymerization processes run slower.

The most stable composites turned out to be those modified with lactic acid oligomer and with stearic acid or ε-caprolactone (Figure 3). The latter blocked the terminal units and there was no lactide formation or modifier degradation.

### 3.5. SEM Investigations

**Figure 4:**
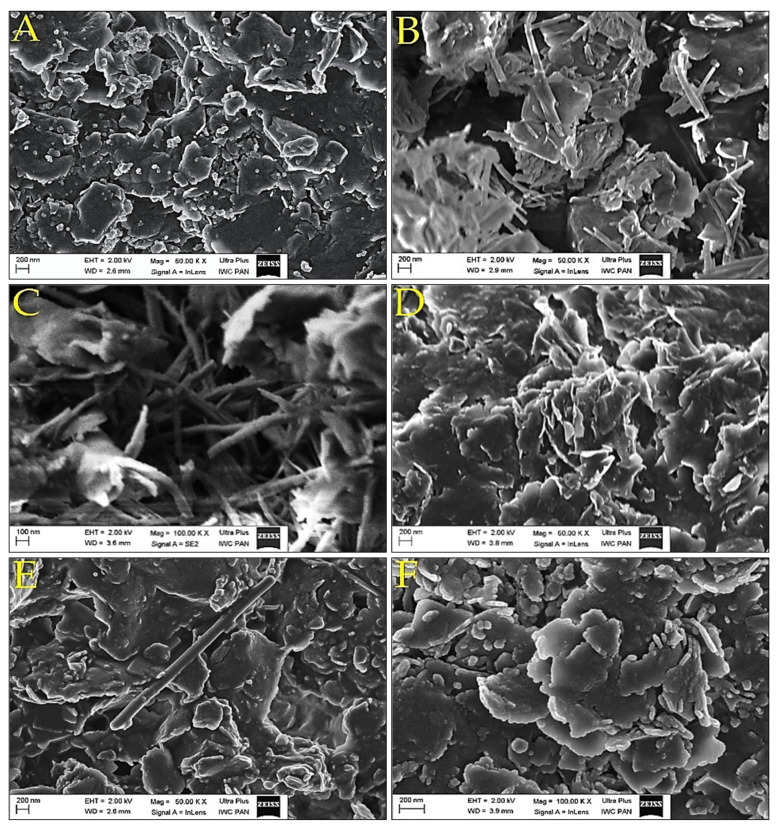
SEM images for montmorillonites: (**A**)—Mt, (**B**)—Mt-LAc, (**C**)—Mt-LAc-CAc, (**D**)—Mt-LAc-MA, (**E**)—Mt-LAc-PT, (**F**)—Mt-LAc-SAc.

The morphology of the sodium Mt and the modified organophilic Mt composites were examined by SEM. SEM images—Figure 4 showed that sodium Mt formed agglomerates composed of plates of irregular shape and a thickness of 20–30 nm.

Samples modified with lactic acid oligomers—Figure 4B had a slightly different morphology to the original Mt. Distinct specks of fibers in irregular shapes with a length of 100 nm can be seen on the surface. Presumably, these could be adsorbed organic material particles or partially deagglomerated filler fragments covered by a layer of organic material. In the SEM image for Mt-LAc-CAc—Figure 4C, the presence of a significant amount of fibrous matter was easily observed, which formed domains between the mineral flakes. Seemingly the presence of citric acid in the reaction mixture and the increased number of end carboxyl groups in the organic material promoted the partial disintegration of the Mt layers and therefore possibly the adsorption of the organic material onto the mineral surface. The Mt-LAc-MA composite—Figure 4D, formed dense porous agglomerates with dimensions of several dozen micrometers. The SEM image of the Mt-LAc-PT composite—Figure 4E, showed that the Mt formed agglomerate plates with a size of several micrometers, among which single fibers with a diameter of about 100 nm could also be observed. Although, their number was significantly smaller than the amount seen in composites with linear oligomers generated in situ. The SEM image of Mt-LAc-SAc—Figure 4F did not show the formation of a new phase on the flakes of modified Mt, other than those already present for Mt-modified with lactic acid oligomer. However, this does not exclude the possibility of deposition of amorphous organic material on the surface of the flakes.

### 3.6. Swelling Capacity

The water swelling study, Table 1, showed that a sample of unmodified Mt, pre-dried for 3 h at 110 °C, absorbed approximately 5.6 g of water per 1 g of substrate after 72 h of storage in water at room temperature. Water absorption tests of Mt-LAc samples showed that they were able to absorb about 2.3 g of water per 1 g of sample at room temperature, i.e., about 2.5 times less than the starting Mt. The lactic acid-citric acid copolymer composites were able to absorb more water (3.2 g/g), but still significantly less than the starting Mt samples (~5.6 g/g). Thus, it can be assumed that an increase in the content of carboxyl end groups increased the organic material’s affinity for Mt, but had no significant effect on the formation of bonds with water. In the Mt-LAc-PT composite, after a standard drying time of several hours at 110 °C, the sample stored in air lost about 13% of its weight, indicating that the content of weakly bound water molecules in this composite was similar to the starting Mt. The ability to absorb subsequent molecules in the aqueous environment was about 2.8 g/g, which was half that of Mt and slightly higher than for the composite modified with a linear polymer (~2.3 g/g). In the case of the Mt-LAc-MA composite, the water swelling capacity of this sample (~3.1 g/g) was slightly higher than for composites with pentaerythritol modified organic material, but still lower than for unmodified Mt. The introduction of poly(ε-caprolactone) to the composites structure resulted in a significant reduction of the water absorption capacity. The swelling capacity was about 1.5 g/g, so it achieved values similar to composites additionally modified with stearic acid. Mt-LAc-SAc (S_eq_ = 1.356) and Mt-LAc-CAP (S_eq_ = 1.489) as well as Mt-LAc (S_eq_ = 2.297) composites had the lowest swelling capacity and were 2–3 times lower than unmodified sodium Mt (S_eq_ = 5.651).

### 3.7. Possibility to Use Montmorillonites with Lactic Acid Oligomers and Other Environmentally Friendly Compounds to Modify Mechanical Properties of PLA

Tensile strength, relative elongation at break, and Young’s modulus of the cords were tested to investigate the basic mechanical parameters. The results obtained confirm the literature reports on adverse effects of unmodified Mt on the mechanical properties of PLA. Since it was found that Mt-LAc-CAP is the most thermally stable and has excellent hydrophobic properties, it was chosen to be tested with PLA (PLA-Mt-LAc-CAP). Samples of sodium montmorillonite (PLA-Mt) and lactic acid-modified montmorillonite (PLA-Mt-LAc) were also tested for comparison. Mt modification facilitates distribution of montmorillonite in PLA, no visible filler agglomerates form.

The average linear density of these samples is expressed in tex = 0.001 g/m. The linear density of PLA was 153.13 ± 2.39 [tex] while the linear density for PLA-Mt was 80.83 ± 0.72 [tex]. PLA with the addition of modified montmorillonites had a value for PLA- Mt-LAc of 100.94 ± 0.63 [tex] and for PLA- Mt-LAc-CAP of 81.56 ± 3.23 [tex], respectively. As can be seen from the data presented, samples containing fillers had a significantly lower linear density than unfilled samples.

**Figure 5:**
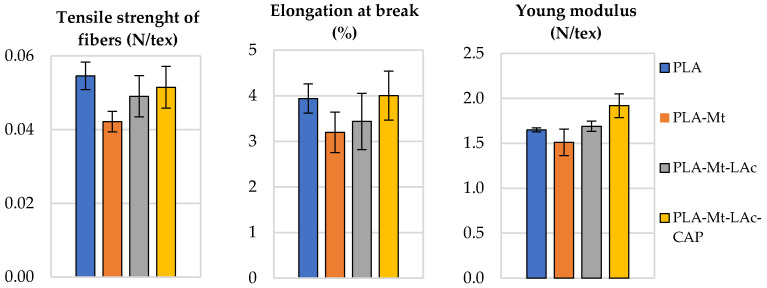
Tensile strength of fibers, elongation at break, Young modulus for PLA, PLA-Mt, PLA-Mt-LAc and PLA-Mt-LAc-CAP.

Figure 5 shows the values of Young’s modulus, tensile strength and relative elongation at break. In this study, Young’s modulus and tensile strength were specified in N/tex because the lines were tested.

As can be seen from the presented data, addition of unmodified Mt causes a decrease in Young’s modulus and tensile strength, and elongation at break. Introduction of Mt modified only with lactic acid oligomers does not cause any changes in Young’s modulus of PLA, while tensile strength and elongation at break are practically unchanged compared to PLA without filler. In the case of the sample containing Mt modified with lactic acid oligomers and ε-caprolactone, a slight increase in Young’s modulus (about 16%) and elongation at break is observed, while tensile strength remains practically unchanged compared to PLA without filler. Based on the determined standard deviations for the individual quantities (tensile strength of fibres, elongation at break, and Young modulus), it can be concluded that most of the determined parameters do not differ significantly from each other.

These results may indicate that structural disorders are formed at the Mt/PLA inter-face, which decrease the mechanical parameters of the whole system. The presence of lac-tic acid oligomers on the Mt surface improves the compatibility of the filler with polymeric matrix; however, it does not lead to significant improvement of mechanical properties. It can therefore be assumed that under the processing conditions Mt modified with lactic acid oligomers does not exfoliate and does not form a nanocomposite. For the full benefits of the introduction of such modified Mt to PLA, the results of other mechanical and flammability tests are needed. It is estimated that such a modified composite, due to the addition of the filler, will improve the properties associated with the addition of inorganic filler, i.e., better thermal stability and reduced flammability. These advantages can be achieved without deteriorating the mechanical properties of PLA. The results of this research will be presented in the next article.

## 4. Conclusions

A new organophilic montmorillonite was synthesized and it was modified with lactic acid oligomers and other compounds such as citric acid, stearic acid, maleic anhydride, pentaerythritol, and ε-caprolactone. The obtained composites were characterized by means of FTIR spectroscopy, elemental analysis, XRD, thermogravimetric methods, SEM, and swelling capacity determination. The obtained modified composites contained an organic phase, which was confirmed by elemental analysis, FTIR, and SEM spectroscopy. When analyzing the diffractograms, the separation into layers could be seen. The greatest level of delamination occurred for Mt-LAc-MA, where the parameter d_001_ was 2.01 nm, it was the highest value amongst all tested composites. Thermal analyses of modified Mt’s revealed that the most stable composites were those modified with a lactic acid oligomer, stearic acid or ε-caprolactone. They had blocked terminal units. This led to the failure to form lactide. The composites modified with lactic acid oligomers, stearic acid, or ε-caprolactone were also characterized as having the lowest swelling values, and in turn, the highest hydrophobicity.

Montmorillonites have been modified in order to obtain organophilized montmorillonites. The first mechanical tests of PLA and organophilized Mt (PLA-OMt) show promising results. Modification of PLA with Mt-LAc-CAP a slight increase in Young’s modulus (about 16%), and elongation at break is observed, while tensile strength remains practically unchanged compared to PLA without filler. The fillers thus obtained could become a new alternative to the existing toxic and heat-labile, traditionally organophilized montmorillonites. The results of full research on the mechanical properties of PLA-OMt composites will be presented in the next article.

## Figures and Tables

**Table 1:** Elemental analysis, interlayer distance d_(001)_, and swelling capacity (S_eq_) for organophilic Mt.

Sample	Elemental Analysis		S_eq_ (g/g)	d_(001)_ (nm)
	%C	%H		
Mt	0.78	1.56	5.651	1.28
Mt-LAc	17.64	2.77	2.297	1.77
Mt-LAc-CAc	13.23	2.05	3.176	1.91
Mt-LAc-MA	21.43	3.06	3.131	2.01
Mt-LAc-PT	14.81	2.09	2.828	1.83
Mt-LAc-SAc	12.79	2.25	1.356	1.79
Mt-LAc-CAP	19.99	3.37	1.489	1.75

S_eq_ (g/g)—swelling capacity; d_(001)_ (nm)—interlayer distance.

## Data Availability

All experimental data to support the findings of this study are available upon request by contacting the corresponding authors.

## Supplementary Materials

The following are available online at https://www.mdpi.com/article/10.3390/ma14216286/s1, Figure S1: Flow chart of the procedure applied during the synthesis of pure LAc oligomer (oligoLAc)., Figure S2: Flow chart of the procedure applied during the modification of montmorillonite with: lactic acid oligomers (synthesis of Mt-LAc, no Reagent 3 used), lactic acid oligomers and maleic anhydride (synthesis of Mt-LAc-MA, MA used as Reagent 3) or lactic acid oligomers and pentaerythritol (synthesis of Mt-LAc-PT, PT used as Reagent 3)., Figure S3: Flow chart of the procedure applied during the modification of montmorillonite with lactic acid oligomers and stearic acid (synthesis of Mt-LAc-SAc)., Figure S4: Flow chart of the procedure applied during the modification of montmorillonite with lactic acid oligomers and citric acid (synthesis of Mt-LAc-CAc)., Figure S5: Flow chart of the procedure applied during the modification of montmorillonite with lactic acid oligomers and ɛ-caprolactone (synthesis of Mt-LAc-CAP).
