# Supplementary material for: New Organophilic Montmorillonites with Lactic Acid Oligomers and Other Environmentally Friendly Compounds and Their Effect on Mechanical Properties of Polylactide (PLA)
Source: Materials (Basel). 2021 Oct 21;14(21):6286. doi: 10.3390/ma14216286 (PMC8584992; doi:10.3390/ma14216286)
Supplement: Supplementary file 1 [file materials-14-06286-s001.zip › materials-1415991-supplementary.pdf]

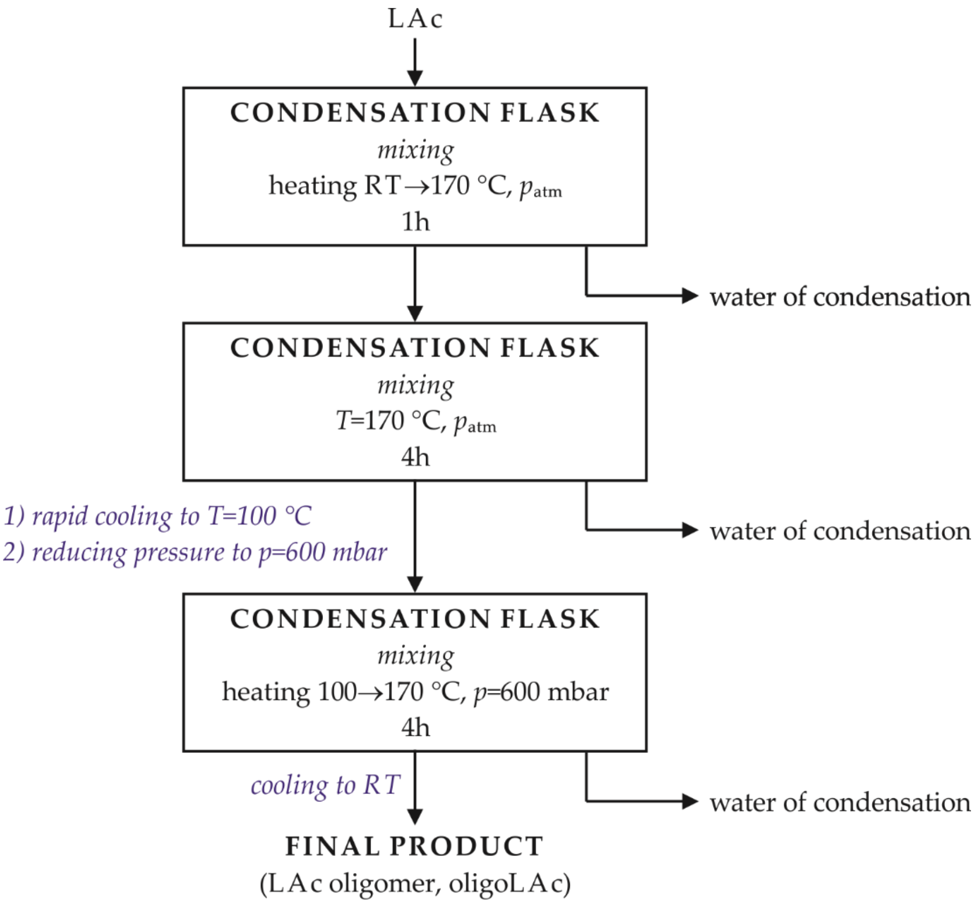

Figure S1. Flow chart of the procedure applied during the synthesis of pure LAc oligomer (oligoLAc). Abbreviations: RT—room temperature, Lac—lactic acid, p_{atm}—atmospheric pressure

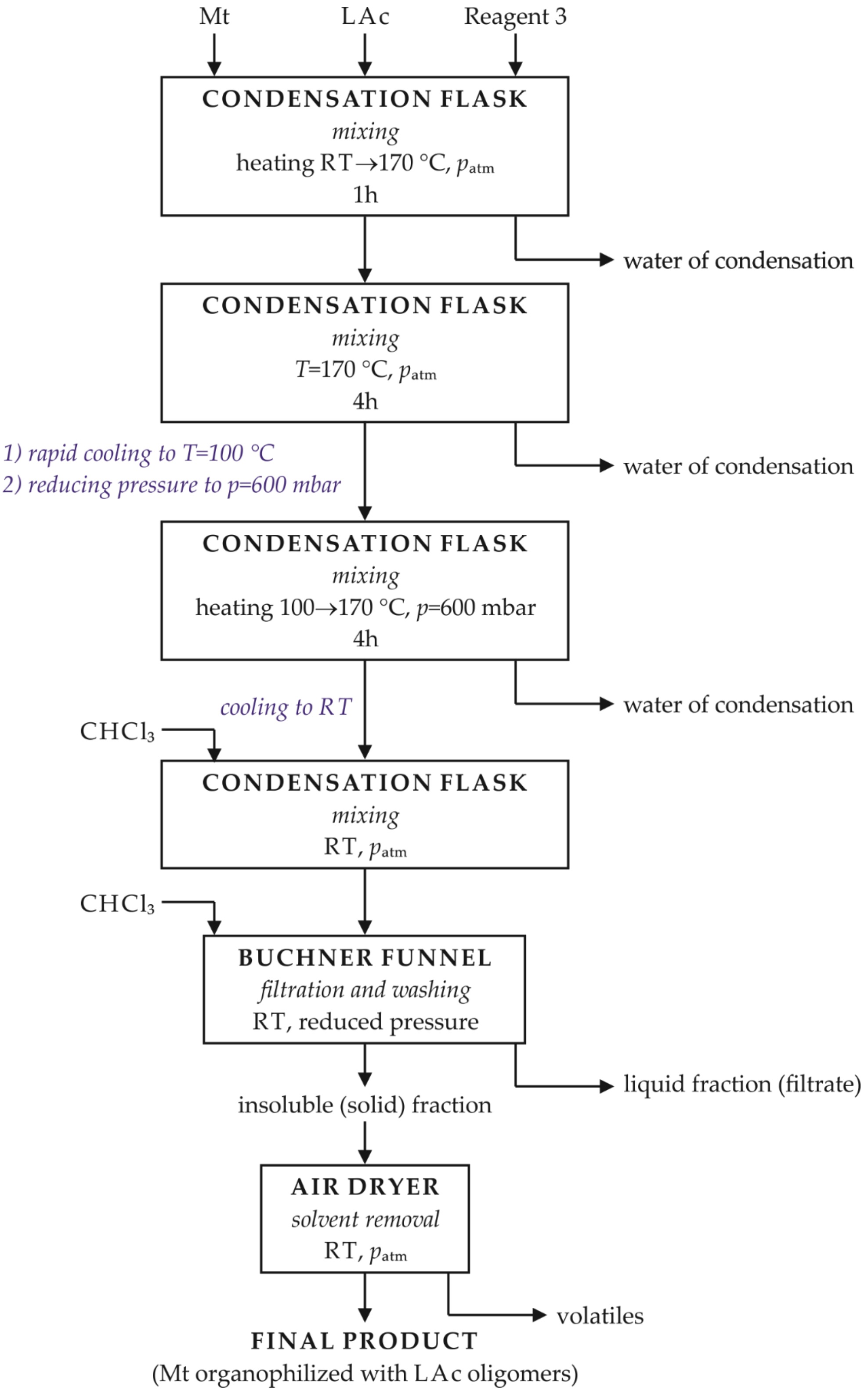

Figure S2. Flow chart of the procedure applied during the modification of montmorillonite with: lactic acid oligomers (synthesis of Mt-LAc, no Reagent 3 used), lactic acid oligomers and maleic anhydride (synthesis of Mt-LAc-MA, MA used as Reagent 3) or lactic acid oligomers and pentaerythritol (synthesis of Mt-LAc-PT, PT used as Reagent 3). Abbreviations: Mt- montmorillonite, LAc—lactic acid, MA—maleic anhydride, PT—pentaerythritol, RT—room temperature, p_{atm}—atmospheric pressure,

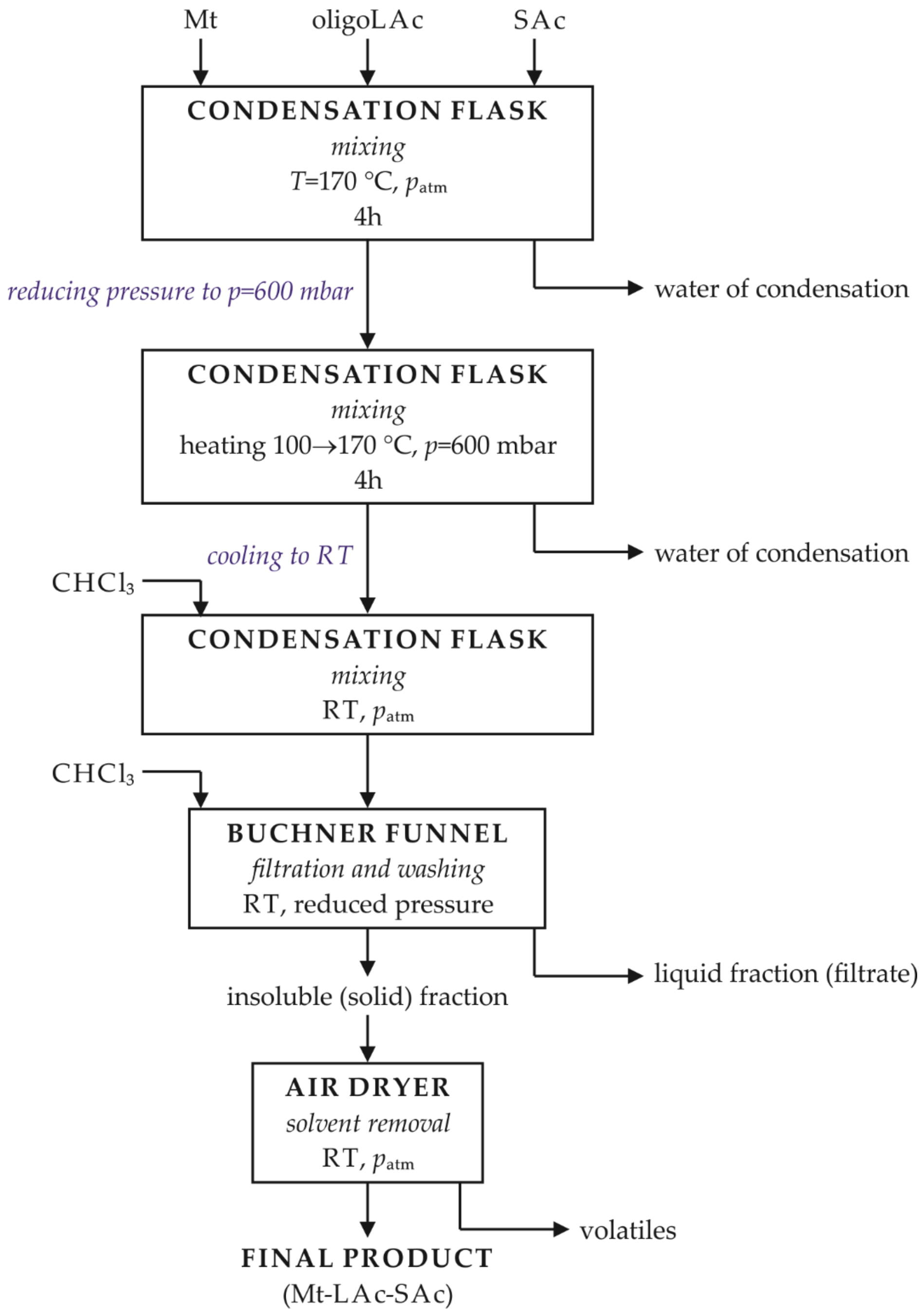

Figure S3. Flow chart of the procedure applied during the modification of montmorillonite with lactic acid oligomers and stearic acid (synthesis of Mt-LAc-SAc). Abbreviations: Mt-montmorillonite, oligoLAc—lactic acid oligomer, SAc—stearic acid, p_{atm}—atmospheric pressure, RT—room temperature,.

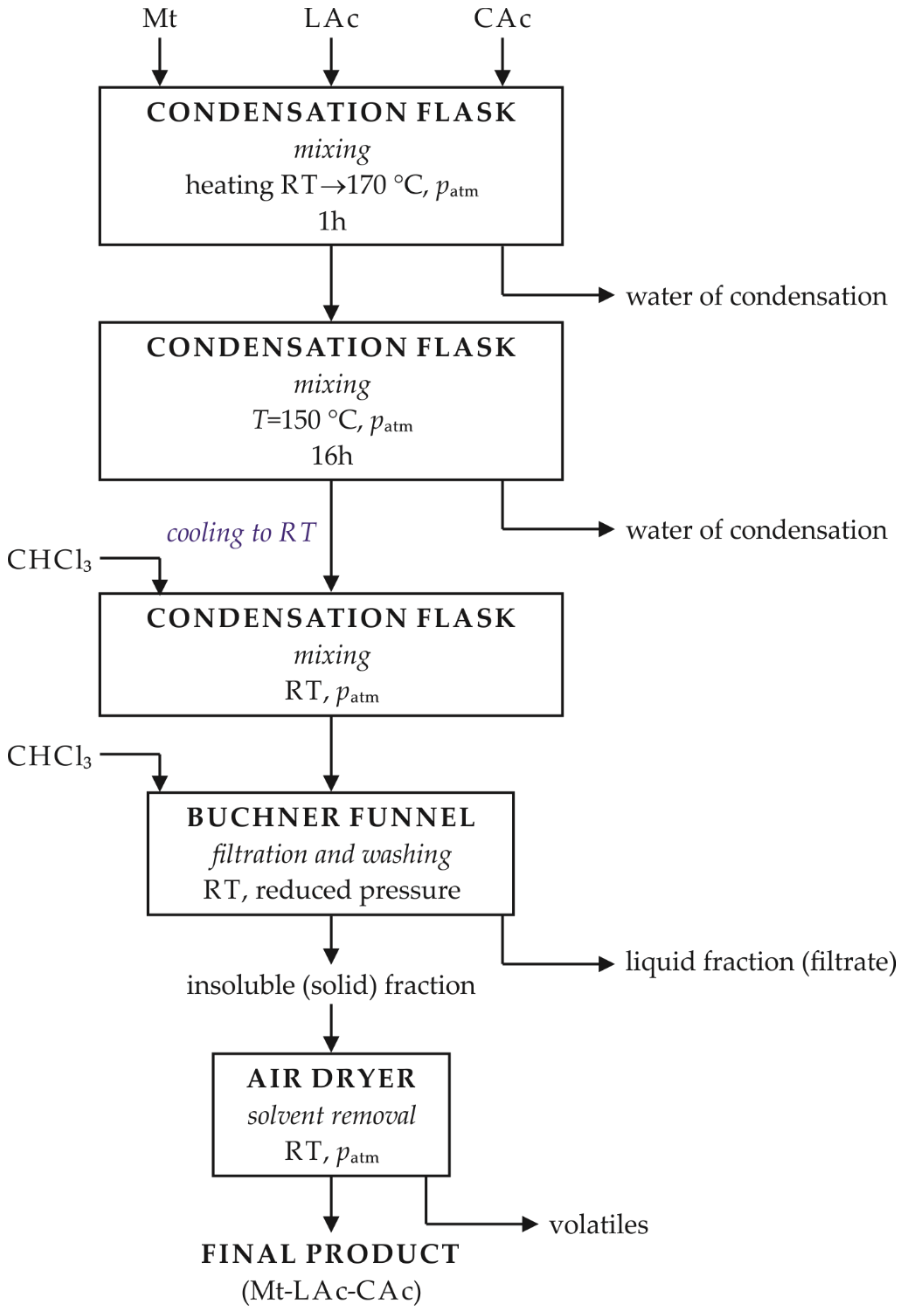

Figure S4. Flow chart of the procedure applied during the modification of montmorillonite with lactic acid oligomers and citric acid (synthesis of Mt-LAc-CAc). Abbreviations: Mt-montmorillonite, Lac—lactic acid, CAc—citric acid, RT—room temperature, p_{atm}—atmospheric pressure.

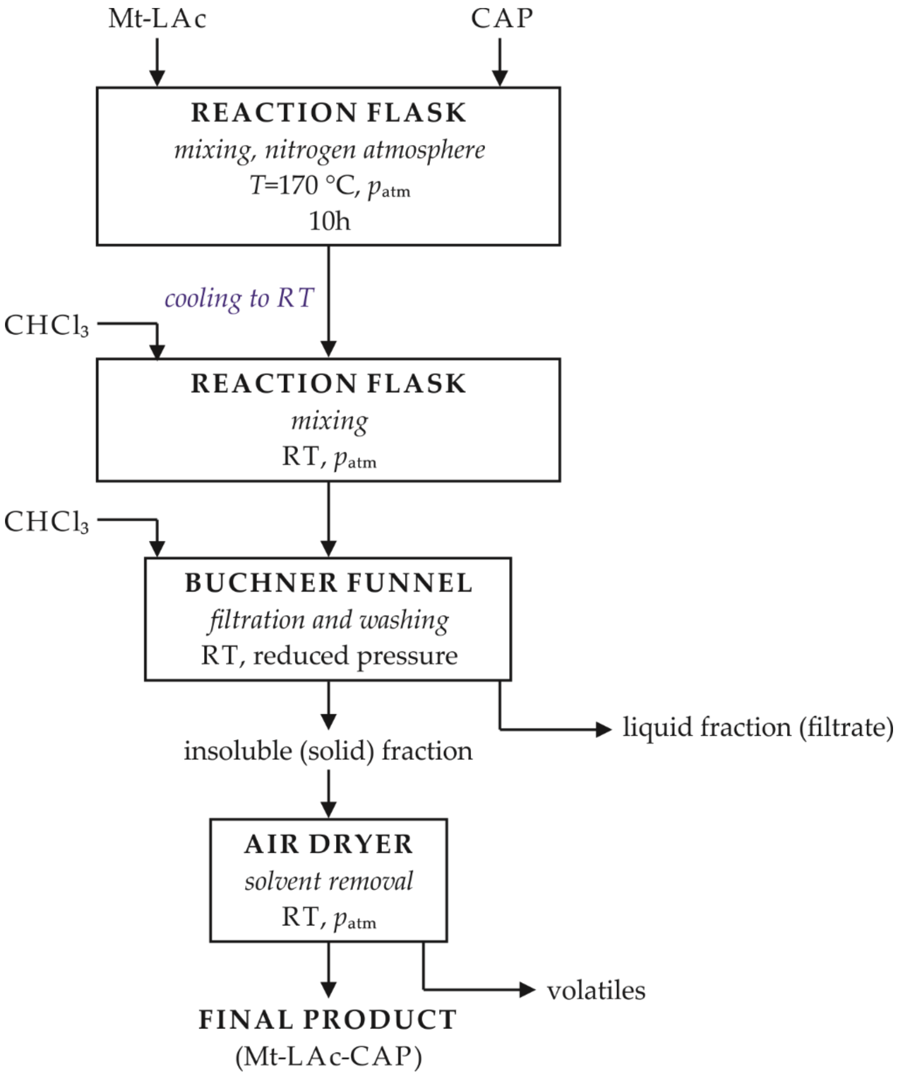

Figure S5. Flow chart of the procedure applied during the modification of montmorillonite with lactic acid oligomers and ε-caprolactone (synthesis of Mt-LAc-CAP). Abbreviations: Mt-montmorillonite, LAc—lactic acid, CAP—ε-caprolactone, RT—room temperature, p_{atm}—atmospheric pressure.
